# Inhibition of Autophagy by Berbamine Hydrochloride Mitigates Tumor Immune Escape by Elevating MHC-I in Melanoma Cells

**DOI:** 10.3390/cells13181537

**Published:** 2024-09-13

**Authors:** Jinhuan Xian, Leilei Gao, Zhenyang Ren, Yanjun Jiang, Junjun Pan, Zheng Ying, Zhenyuan Guo, Qingsong Du, Xu Zhao, He Jin, Hua Yi, Jieying Guan, Shan Hu

**Affiliations:** 1 Research Center of Integrative Medicine, School of Basic Medical Sciences, Guangzhou University of Chinese Medicine, Guangzhou 510006, China; jinhuanxian@stu.gzucm.edu.cn (J.X.); gaoleilei@stu.gzucm.edu.cn (L.G.); renzhenyang@stu.gzucm.edu.cn (Z.R.); panjunjun1@oncolab.cn (J.P.); yingzheng@oncolab.cn (Z.Y.); guozhenyuan@oncolab.cn (Z.G.); duqingsong@oncolab.cn (Q.D.); zhaoxu@oncolab.cn (X.Z.); jinhe188@gzucm.edu.cn (H.J.); yingying020@gzucm.edu.cn (H.Y.); 2 Department of Pathology and Pathophysiology, Guangzhou University of Chinese Medicine, Guangzhou 510006, China; 3 Department of Biochemistry, Guangzhou University of Chinese Medicine, Guangzhou 510006, China; 4 Department of Anaesthesia and Intensive Care, The Chinese University of Hong Kong, Hong Kong 999077, China; 1155149754@link.cuhk.edu.hk

**Keywords:** MHC-I, antigen presentation, tumor immune escape, Berbamine hydrochloride, autophagy

## Abstract

Impaired tumor cell antigen presentation contributes significantly to immune evasion. This study identifies Berbamine hydrochloride (Ber), a compound derived from traditional Chinese medicine, as an effective inhibitor of autophagy that enhances antigen presentation in tumor cells. Ber increases MHC-I-mediated antigen presentation in melanoma cells, improving recognition and elimination by CD8^+^ T cells. Mutation of Atg4b, which blocks autophagy, also raises MHC-I levels on the cell surface, and further treatment with Ber under these conditions does not increase MHC-I, indicating Ber’s role in blocking autophagy to enhance MHC-I expression. Additionally, Ber treatment leads to the accumulation of autophagosomes, with elevated levels of LC3-II and p62, suggesting a disrupted autophagic flux. Fluorescence staining and co-localization analyses reveal that Ber likely inhibits lysosomal acidification without hindering autophagosome–lysosome fusion. Importantly, Ber treatment suppresses melanoma growth in mice and enhances CD8^+^ T cell infiltration, supporting its therapeutic potential. Our findings demonstrate that Ber disturbs late-stage autophagic flux through abnormal lysosomal acidification, enhancing MHC-I-mediated antigen presentation and curtailing tumor immune escape.

## 1. Introduction

Melanoma, a malignant skin cancer originating from melanocytes, is noted for its aggressive nature and poor prognosis. The global incidence of melanoma has been on the rise, with approximately 325,000 new cases reported worldwide in 2020 [1]. Additionally, lifestyle trends such as tanning and tattooing are contributing to a decreasing age of onset among populations [1]. Traditional treatment modalities, including surgery, chemotherapy, radiotherapy, and cytokine therapy, have demonstrated limited effectiveness against metastatic melanoma, leaving the five-year survival rate for these patients unsatisfactorily low [2]. This underscores an urgent demand for novel therapeutic interventions.

The landscape of melanoma treatment has been revolutionized by advancements in immunotherapy, significantly improving patient survival rates in recent years [3]. Tumor immunotherapy harnesses specific immune responses, particularly the activation of cytotoxic T cells (CD8^+^ T cells). These cells recognize and eliminate tumor cells that present antigens via major histocompatibility complex class I (MHC-I), thereby enhancing the efficacy of the treatment [4]. The antigen presentation by MHC-I molecules in cancer cells is critical in this process, and inefficiencies in this mechanism can facilitate tumor immune evasion [5]. Consequently, understanding the pathways that impair tumor antigen presentation is essential for the development of effective melanoma therapies.

Recent research has revealed that autophagy, a cellular degradation process, plays a crucial role in tumor immune evasion [6]. Autophagy not only controls the degradation of immune checkpoint proteins in cancer cells, but also affects antigen presentation and delivery by antigen-presenting cells, as well as modulates T cell activity [7,8]. Given these multifaceted roles, inhibiting autophagy could disrupt these tumor-protective mechanisms, potentially enhancing the immune system’s ability to recognize and destroy cancer cells. Moreover, reduced autophagy has been associated with an increase in reactive oxygen species within cells, leading to heightened susceptibility to apoptosis [9]. Therefore, targeting autophagy presents a promising strategy to amplify the effectiveness of cancer immunotherapy.

Currently, autophagy inhibitors are divided into two categories based on their mechanism of action. Early-stage inhibitors, such as 3-methyladenine (3-MA) and wortmannin, disrupt the initial formation of autophagosome membranes [10,11]. Late-stage inhibitors, including bafilomycin A1 (Baf) and chloroquine (CQ), obstruct the fusion of autophagosomes with lysosomes and inhibit lysosomal acidification [12,13]. Despite their potential, the clinical application of these inhibitors is often restricted due to their high toxicity [14]. Therefore, the development of safer, more effective autophagy inhibitors remains a critical clinical challenge.

Chinese herbal medicines, known for their vast array of bioactive properties, are increasingly recognized in drug development across various therapeutic areas. Berbamine hydrochloride (Ber), a natural monomeric compound extracted from Chinese medicinal herbs such as Coptis chinensis (Chinese goldthread) and Berberis spp. (barberry), exemplifies this potential. Historically noted for its potent antibacterial properties, Ber has shown considerable therapeutic effectiveness against diverse infectious diseases [15,16]. Clinically, it is predominantly utilized to elevate peripheral white blood cell counts [17]. Recent studies also highlight its anticancer capabilities [18], although the mechanisms underlying these effects warrant further exploration.

In our investigation, we identify Ber as a potent late-stage autophagy inhibitor that mitigates tumor immune evasion in melanoma. We demonstrate that Ber disrupts autophagy in melanoma cells by impairing lysosomal acidification, thereby enhancing MHC-I-mediated antigen presentation. This action significantly boosts the efficacy of CD8^+^ T cell-mediated tumor clearance, offering a promising avenue to reinforce anti-tumor immune responses in melanoma.

## 2. Materials and Methods

### 2.1. Cell Culture

B16-F10 (referred to as B16) melanoma cells were procured from the American Type Culture Collection (ATCC, Rockville, MD, USA), and A375 *human* melanoma cells were sourced from Wuhan Pricella Biotechnology (Wuhan, China). B16 cells stably expressing ovalbumin (OVA) were acquired from Beijing Crispr Biotechnology Co., Ltd. (Beijing, China). B16 cells were cultured in RPMI-1640 medium enriched with 10% fetal bovine serum (FBS), as reported in other publications [19,20], while A375 cells were maintained in Dulbecco’s modified Eagle’s medium (DMEM, Gibco, Grand Island, NY, USA) supplemented with 10% FBS. A375 cells stably expressing GFP-tagged LC3 (A375-GFP-LC3) were cultured under identical conditions to their wild-type counterparts. To induce autophagy via nutrient deprivation, A375-GFP-LC3 cells were washed with phosphate-buffered saline (PBS) and incubated in Hank’s Balanced Salt Solution (HBSS) for 6 h. All cells were incubated at 37 °C in a humidified atmosphere containing 5% CO_2_.

### 2.2. Reagents and Antibodies 

Berbamine hydrochloride (Ber, ≥99% purity, Catalog No. T2920) was sourced from TargetMol Chemicals Inc. (Shanghai, China). Autophagy inhibitors bafilomycin A1 (#S1413) and chloroquine (Catalog No. C6628) were obtained from Selleckchem (Houston, TX, USA). Primary antibodies targeting β-actin (#3700), LC3-I/II (#12741), p62 (#88588), cathepsin B (CatB, #31718), and cathepsin D (CatD, #2284) were purchased from Cell Signaling Technology (Boston, MA, USA). The primary antibody against HLA-A/B (A8754) was supplied by ABclonal (Wuhan, China), the anti-CD8a (#14-0081-85) antibody was purchased from Thermo Fisher Scientific (Waltham, MA, USA), and the H-2K^b^ (*mouse* MHC class I) antibody (#sc-59199) was obtained from Santa Cruz (Dallas, TX, USA). Secondary antibodies, HRP-conjugated anti-*mouse* IgG (#AS004), anti-*rat* IgG (#AS0028), and anti-*rabbit* IgG (#AS014) were procured from ABclonal (Wuhan, China); Alexa Fluor^®^ 594-conjugated anti-*rat* IgG was obtained from Abcam (#GR3438930-1, Cambridge, UK), and Alexa Fluor^®^ 594-conjugated anti-*rabbit* IgG (#8889) and Alexa Fluor^®^ 488-conjugated anti-*rabbit* IgG (#4412) were supplied by Cell Signaling Technology (Boston, MA, USA).

### 2.3. Western Blot Analysis 

Post-treatment, cells were lysed using a buffer supplemented with protease inhibitors. Protein concentrations were determined using the BCA Protein Assay Kit (#MA0082, Meilunbio, Dalian, China). The samples (20 µg protein each) were separated by SDS-PAGE and transferred onto PVDF membranes. The membranes were blocked with 5% skim milk in TBST, incubated overnight at 4 °C with primary antibodies (dilution 1:1500) and then with horseradish peroxidase-conjugated secondary antibodies for one hour at room temperature. Protein bands were visualized using the Tanon™ 5200CE Chemi-Image System (Tanon, Shanghai, China).

### 2.4. In Vitro Cytotoxicity Assay

OT-I mice, harboring T cell receptors specific to OVA_257-264 presented by H-2K^b^, were sourced from GENEANDPEASE (Yangzhou, China). Their spleens were harvested, mechanically dissociated, and lymphocytes were isolated using a lymphocyte separation solution (#7211011, Dakewe, Shenzhen, China). CD8^+^ T cells were enriched by incubating with *mouse* CD8a (Ly-2) MicroBeads (#130-117-044, Miltenyi Biotec, Bergisch Gladbach, Germany) and MACS buffer (#130-091-222-1, Miltenyi Biotec) at 4 °C for 10 min, followed by separation using a magnetic column. The cells were subsequently resuspended in complete medium enriched with IL-2 (#212-12-100, Peprotech, Cranbury, NJ, USA) for further experiments.

B16-OVA cells were seeded in 12-well plates overnight and subsequently treated with varying concentrations of Ber. After treatment, the drug was washed out, and the cells were co-cultured with CD8^+^ T cells derived from OT-I mice (referred to as OT-I T cells) for 24 h. Post-co-culture, supernatants were collected for IFN-γ quantification using an ELISA kit (#RK00019, ABclonal, Wuhan, China). Simultaneously, co-cultured cells were washed with PBS to remove OT-I T cells. The remaining B16-OVA cells were then fixed with 4% paraformaldehyde, washed twice with PBS, and stained with crystal violet for 10 min. After extensive rinsing with PBS, the viability of tumor cells was quantified by measuring the optical density at 570 nm following crystal violet staining. To assess T cell-mediated cytotoxicity following Ber treatment, co-cultured OT-I T cells and Ber-pre-treated B16-OVA cells were harvested and subjected to dual staining with Fixable Viability Stain 620 (#553142, BD Biosciences, San Jose, CA, USA) and APC-conjugated anti-*mouse* CD8a (#100712, Biolegend, San Diego, CA, USA). Flow cytometry analysis was performed to identify the apoptotic B16-OVA cells, which were characterized as CD8a-negative and FVS620-positive, indicative of T cell-mediated tumor cell killing.

### 2.5. Flow Cytometry Analysis

Murine or human tumor cells, treated with Ber for designated time intervals, were harvested by trypsinization and washed with PBS containing 2% FBS. For immunophenotyping, the cells were stained in the dark at room temperature for 20 min using the following antibodies: Pacific Blue™ anti-*mouse* H-2K^b^ (#116514, BioLegend, San Diego, CA, USA), FITC anti-*mouse* H-2K^b^ (#553569, BD Biosciences, San Jose, CA, USA), and the OVA257-264 peptide bound to H-2K^b^ (#141605, BioLegend). After staining, the cells were washed twice, resuspended in a buffer, filtered through a 70 µm nylon mesh, and immediately analyzed using a BD LSR Fortessa flow cytometer. Data analysis was conducted using Flow Jo software (version. 10.8.1; Tree Star Inc., Ashland, OR, USA).

### 2.6. Immunofluorescence Assay

Cells were plated on coverslips in 12-well plates and treated with Ber for 24 h. Following a PBS wash, the cells were fixed with 4% paraformaldehyde for 20 min at room temperature. Blocking was performed for two hours with 5% fetal bovine serum in PBS. The cells were then incubated overnight at 4 °C with a primary antibody specific to HLA-A/B or LC3-I/II. After washing with PBS, the cells were incubated with Alexa Fluor^®^ 594-conjugated anti-*rabbit* IgG or Alexa Fluor^®^ 488-conjugated anti-*rabbit* IgG for one hour at room temperature, followed by staining with Hoechst 33342 (5 µg/mL) for 10 min. Slides were mounted using a Prolong™ Diamond Antifade Mountant (#P36970, Invitrogen, Carlsbad, CA, USA) and imaged with an LSM 800 confocal microscope (Carl Zeiss, Jena, Germany). For immunofluorescence staining of paraffin sections, sections underwent antigen retrieval and blocking prior to overnight incubation with an antibody against CD8a. They were then incubated with Alexa Fluor^®^ 594-conjugated anti-*rat* IgG for one hour at room temperature, counterstained with DAPI, and analyzed with a confocal microscope.

### 2.7. Lentivirus Production and Infection 

Lentiviral vectors were transfected into HEK293FT cells using Polyethylenimine Linear (PEI) MW40000 (#40816ES02, Yeasen, Shanghai, China) in Opti-MEM™ (#31985062, Gibco, Grand Island, NY, USA). For lentivirus production, HEK293FT cells were co-transfected with the lentiviral vector and packaging plasmids psPAX2 and pMD2.G at a 1:3:4 ratio. Six hours post-transfection, the medium was replaced with DMEM supplemented with 10% FBS. Viral supernatants were harvested 48 h later, filtered through a 0.22 μm pore-size filter, and used for infecting cells in DMEM supplemented with 10% FBS. GFP-LC3 plasmids were provided by Professor William KK Wu (The Chinese University of Hong Kong), and the pSLIK_Hyg_mTurquoise2_Atg4bC74A plasmids were sourced from Alec Kimmelman’s lab via Addgene (#161733, Watertown, MA, USA). The hLAMP1-mCherry construct (#VB900162-5529yba) was designed and purchased from VectorBuilder (Guangzhou, China).

### 2.8. mCherry-GFP-LC3B Transfection

The pBABE-puro mCherry-GFP-LC3B plasmid (Addgene, #22418) was a gift from Jayanta Debnath [21]. Cells were seeded onto glass coverslips in 12-well plates and transiently transfected with 0.5 µg of the plasmid. After 24 h, the cells were treated with Ber, fixed with 4% paraformaldehyde, and evaluated for autophagy flux using an LSM 800 confocal microscope (Carl Zeiss, Jena, Germany). The number of puncta per cell was counted and compared by researchers who were blinded to the grouping information.

### 2.9. RNA Isolation and Quantitative RT-PCR

Total RNA was extracted using TRIzol reagent (#15596018CN, Thermo Fisher Scientific) following the manufacturer’s guidelines. The RNA concentration was measured, and samples were reverse transcribed into cDNA using an Evo M-MLV RT Kit (#AG11706, Accurate Biology, Changsha, China). mRNA levels were quantified using SYBR-Green qPCR Master Mix (#AG11740, Accurate Biology). The sequences of the qRT-PCR primers can be found in Supplementary Table S1.

### 2.10. LysoTracker Staining

A375 cells were seeded into a confocal dish and incubated for 24 h. On the second day, drug treatment was administered according to the experimental design. After treatment, the medium was discarded, and the cells were washed twice with PBS. Lysosomes were then stained using LysoTracker Red DND-99 (50 nM, L7528; Invitrogen, Carlsbad, CA, USA) following the manufacturer’s instructions. Images were captured using a laser scanning confocal microscope (LSM 800; Carl Zeiss, Jena, Germany) and analyzed using ImageJ software (Version 1.4.3; NIH, Bethesda, MA, USA).

### 2.11. Autophagosome and Lysosome Colocalization

A375 cells stably expressing GFP-LC3 were cultured in confocal dishes and treated with Ber and Chloroquine (CQ) for 24 h. Post-treatment, the cells were washed twice with PBS and stained with LysoBrite™ Red for 20 min at 37 °C in DMEM to label lysosomes. After staining, the cells were washed again with PBS and imaged using an LSM 800 confocal microscope (Carl Zeiss, Jena, Germany).

### 2.12. Electron Microscopy 

Cells were rinsed twice with PBS and collected into a 15 mL centrifuge tube. The cell suspension was centrifuged at 1500–3000 rpm for 5 min. The cell pellets were then fixed with 2.5% glutaraldehyde in the dark at room temperature for 30 min. The fixed cells were mounted on copper grids and examined using a transmission electron microscope.

### 2.13. Immunohistochemistry

Tumor tissues from mice were fixed in 4% paraformaldehyde overnight, embedded in paraffin, and sectioned into 4 µm slices. The sections were deparaffinized, rehydrated, and subjected to antigen retrieval in a boiling citrate buffer (pH 6.0). After blocking with 5% bovine serum albumin, the sections were incubated overnight at 4 °C with anti-CD8a antibody. This was followed by a one-hour incubation with HRP-conjugated anti-*rat* IgG at a 1:200 dilution at room temperature. The stained sections were then imaged using a pathology slide scanner.

### 2.14. In Vivo Analysis

The experiments involving mice were approved by the Animal Ethics Committee at Guangzhou University of Chinese Medicine (Project No. 20240428018). Eight-week-old C57BL/6 mice were purchased from the Guangdong Provincial Medical Laboratory Animal Center and acclimated in a specific pathogen-free (SPF) facility. They were provided with ad libitum access to sterilized water and food. B16 cells (2 × 10^6^ cells in 100 µL PBS) were subcutaneously injected into the right flank of the mice. The mice were then randomly divided into three groups (*n* = 5 per group) and subjected to daily intraperitoneal injections with either vehicle (saline solution), 10 mg/kg Ber, or 20 mg/kg Ber. When the tumor volume approached nearly 800 mm³, the mice were anesthetized, and the tumors were dissected and weighed.

### 2.15. Cell Counting Kit-8 (CCK-8) Assay

To investigate the effect on the proliferation of B16 cells, the cells were seeded at a density of 10,000 cells per well in a 96-well plate. Then, the corresponding drugs were added to the cells according to the experimental protocol. Cell Counting Kit-8 (MA0218, Meilunbio, Dalian, China) was used to examine the proliferation of B16 cells, following the manufacturer’s protocol.

### 2.16. Statistical Analysis

Statistical analyses were conducted using GraphPad Prism version 6 (GraphPad Software, San Diego, CA, USA). The data are presented as the mean ± standard deviation (SD) or standard error of the mean (SEM). Differences between groups were assessed using Student’s *t*-test, one-way ANOVA with Dunnett’s or Tukey’s multiple comparisons test, or two-way ANOVA with Tukey’s multiple comparisons test. Statistical significance was defined as * *p* < 0.05, ** *p* < 0.01, and *** *p* < 0.001; ns indicates not significant.

## 3. Results

### 3.1. Ber Enhances MHC-I-Mediated Antigen Presentation in Melanoma Cells

**Figure 1:**
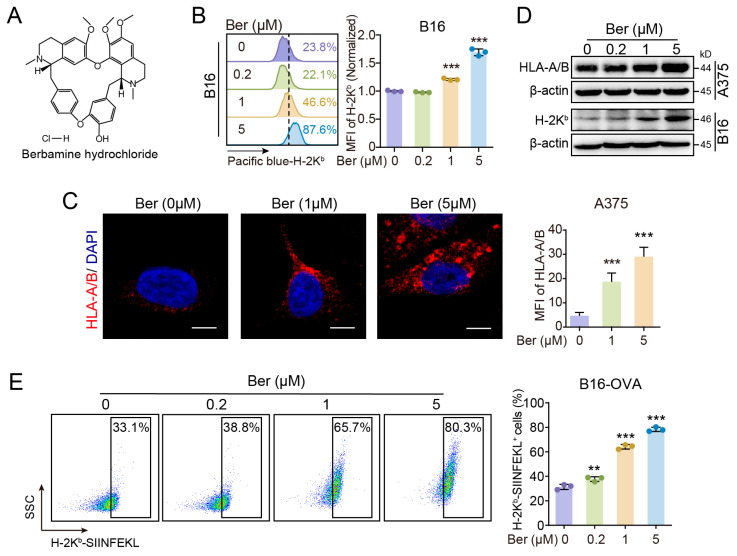
Ber enhances MHC-I-mediated antigen presentation in melanoma cells. (**A**) Chemical structure of Ber. (**B**) Flow cytometry analysis showing the mean fluorescence intensity (normalized) of surface MHC-I (H-2K^b^) on B16 melanoma cells after treatment with indicated concentrations of Ber. Histograms represent a representative experiment. (**C**) Immunofluorescence images of A375 cells treated with Ber, showing an increase in the total number of HLA-positive signals (red fluorescence). Scale bars represent 20 μm. (**D**) Western blot analysis of MHC-I protein levels (*human*, HLA-A/B; *mouse*, H-2K^b^) in melanoma cells under various conditions. Treatments included control (no Ber) and Ber at concentrations of 0.2 μM, 1 μM, and 5 μM. (**E**) Quantification of cell-surface expression of H-2K^b^-SIINFEKL complex on B16 cells by flow cytometry. The graph on the right displays the percentage of H-2K^b^-SIINFEKL-positive cells. ** *p* < 0.01, *** *p* < 0.001 indicate levels of statistical significance.

Ber is a natural monomer derived from traditional Chinese medicinal herbs, such as Coptis chinensis (Chinese goldthread) and Berberis spp. (barberry). The molecular structure of Ber is depicted in Figure 1A. The downregulation of MHC class I molecules on tumor cells contributes significantly to immune evasion [22]. To assess the effects of Ber on MHC-I expression, we treated the *human* melanoma cell line A375, which expresses HLA-A/B as MHC-I, and the *mouse* melanoma cell line B16, which expresses H-2K^b^ as MHC-I, with various concentrations of Ber for 24 h. Flow cytometric analysis demonstrated that Ber treatment increased the surface expression of MHC-I in a concentration-dependent manner in B16 cells (Figure 1B). Similarly, immunofluorescence staining showed a significant increase in HLA-A/B expression in A375 cells post-Ber treatment (Figure 1C). Western blot analysis further confirmed that Ber treatment resulted in a concentration-dependent increase in the intracellular abundance of MHC-I proteins in both A375 and B16 cell lines (Figure 1D). To evaluate the impact of Ber on antigen presentation efficiency, we analyzed the presentation of the fluorescently labeled artificial antigen complex H-2K^b^-SIINFEKL in B16-OVA cells. SIINFEKL is a peptide derived from ovalbumin (OVA). After treating B16-OVA cells with varying concentrations of Ber for 24 h, we observed enhanced expression of the surface H-2K^b^-SIINFEKL complex via flow cytometry (Figure 1E). These findings suggest that Ber effectively increases MHC-I-mediated antigen presentation by upregulating surface and intracellular MHC-I levels in melanoma cells.

### 3.2. Ber Enhances CD8^+^ T Cell-Mediated Cytotoxicity against Melanoma Cells

**Figure 2:**
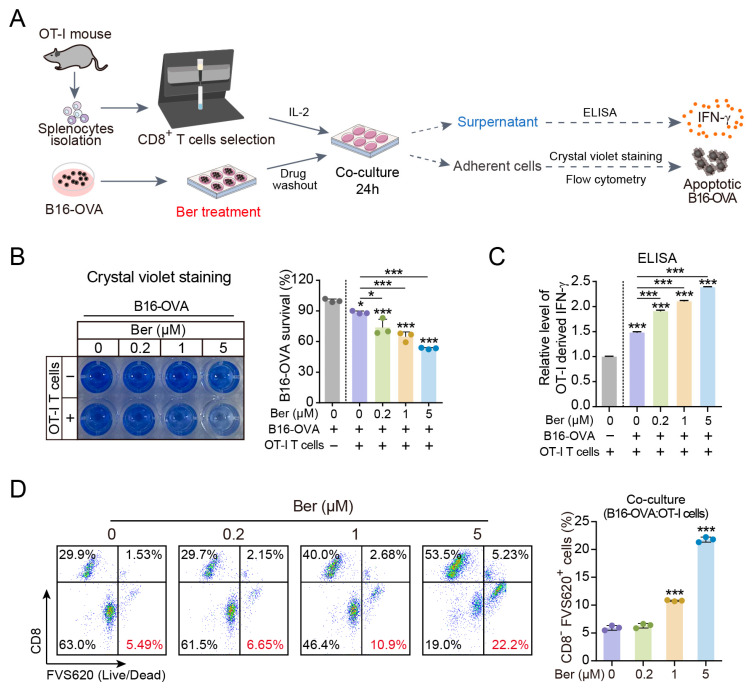
Ber potentiates the cytotoxicity of CD8^+^ T cells against melanoma cells. (**A**) Schematic representation of the co-culture setup between CD8^+^ T cells and B16-OVA melanoma cells. (**B**) Analysis of cell proliferation in B16-OVA cells treated with specified concentrations of Ber and co-cultured with CD8^+^ T cells, assessed using a crystal violet staining assay. (**C**) Quantification of IFN-γ production in the co-cultures of CD8^+^ T cells and B16-OVA cells, measured by ELISA. (**D**) Following treatment with Ber, B16-OVA cells were co-cultured with CD8^+^ T cells for 48 h; apoptotic cells were then quantified by flow cytometry. * *p* < 0.05, *** *p* < 0.001 indicate levels of statistical significance.

CD8^+^ T cells target and eliminate tumor cells presenting specific antigens. To determine whether Ber augments the cytolytic activity of T cells through enhanced antigen presentation, B16-OVA melanoma cells were treated with varying concentrations of Ber for 24 h. These cells were then co-cultured with CD8^+^ T cells from OT-I mice, which recognize the ovalbumin (OVA) antigen, for an additional 24 h (Figure 2A). Crystal violet staining revealed that the cytolytic activity of CD8^+^ T cells increased in a concentration-dependent manner with Ber treatment (Figure 2B). Interferon-gamma (IFN-γ), a critical cytokine for tumor cell recognition and elimination by CD8^+^ T cells, was measured in the co-culture supernatants. Ber treatment enhanced IFN-γ production dose-dependently (Figure 2C). Additionally, flow cytometry analysis was performed to quantify apoptotic tumor cells within the co-culture. The cells were stained with CD8 and FVS620, a dye that penetrates dead cells and binds to lipid membranes, emitting a strong red fluorescence. A progressive increase in the proportion of apoptotic tumor cells (CD8^−^/FVS620^+^) with escalating doses of Ber was observed (Figure 2D). To exclude the influence of Ber on cellular viability, we conducted a CCK8 assay and confirmed that Ber (0, 0.2, 1, 5 µM) did not impact the viability of B16 cells (Figure S2). These findings indicate that Ber enhances MHC-I-mediated antigen presentation, thereby improving the efficacy of CD8^+^ T cells in recognizing and eliminating melanoma cells.

### 3.3. Ber Increases MHC-I Levels in Melanoma Cells by Inhibiting Autophagy

**Figure 3:**
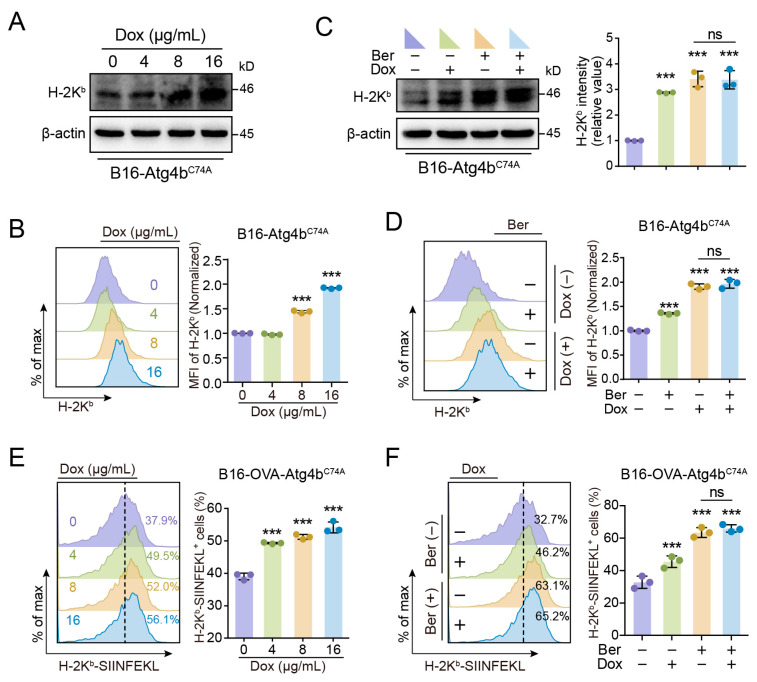
Ber enhances MHC-I levels in melanoma cells by inhibiting autophagy. (**A**) Western blot analysis showing concentration-dependent effects of doxycycline (Dox) on H-2K^b^ protein expression in B16 melanoma cells harboring a Dox-inducible mTurquoise2-Atg4b(C74A) construct. Dox induces autophagy inhibition in this cell line, enabling the study of autophagy’s role in MHC-I expression. (**B**) Flow cytometric analysis of cell surface H-2K^b^ levels in B16 cells expressing Dox-inducible mTurquoise2-Atg4b(C74A) following treatment with a gradient concentration of Dox. (**C**) Western blot assay demonstrating protein expression levels of H-2K^b^ in B16-OVA cells carrying Dox-inducible mTurquoise2-Atg4b(C74A) treated with Ber (5 µM), Dox (8 µg/mL), or their combination for 24 h. (**D**) Quantification of surface MHC-I (H-2K^b^) levels in the same cell lines under the same treatment conditions as in (**C**). (**E**) Quantification of cell-surface expression of the H-2K^b^-SIINFEKL complex on B16-OVA cells with Dox-inducible mTurquoise2-Atg4b(C74A) treated with a gradient concentration of Dox, assessed by flow cytometry. (**F**) Flow cytometric analysis of cell-surface expression of the H-2K^b^-SIINFEKL complex in B16-OVA cells with Dox-inducible mTurquoise2-Atg4b(C74A) following treatment with Ber (5 µM), Dox (8 µg/mL), or their combination for 24 h. *** *p* < 0.001 indicate levels of statistical significance, ns, not significant.

While investigating the mechanisms underlying the increase in MHC-I expression induced by Ber, we first determined whether this was due to enhanced transcription. Our results showed that Ber had no significant effect on MHC-I transcription in either A375 or B16 cells (Figure S1). This led us to hypothesize that Ber enhances MHC-I-mediated tumor antigen presentation through the inhibition of autophagy. To test this hypothesis, we utilized a doxycycline (Dox)-inducible Atg4b mutation in B16 cells, where Atg4b plays a critical role in autophagosome formation and degradation. Western blot analysis showed a significant, dose-dependent increase in MHC-I (H-2K^b^) levels following 24 h of autophagy inhibition with Dox (Figure 3A). Flow cytometry further confirmed a significant increase in surface MHC-I (H-2K^b^) levels after blocking autophagy with the mutated Atg4b and higher Dox concentrations (Figure 3B).

Moreover, when autophagy was inhibited by mutating Atg4b and cells were subsequently treated with Ber, there was no further increase in MHC-I (H-2K^b^) levels (Figure 3C,D). Additionally, analysis of fluorescently labeled H-2K^b^-SIINFEKL demonstrated increased surface expression following treatment with varying concentrations of Dox, whereas simultaneous treatment with Ber and Dox did not further enhance the levels of H-2K^b^-SIINFEKL (Figure 3E,F). These findings suggest that Ber’s enhancement of MHC-I expression and subsequent improvement in antigen presentation in melanoma cells primarily operates through the inhibition of autophagy.

### 3.4. Ber Inhibits Autophagic Flux in Melanoma Cells

**Figure 4:**
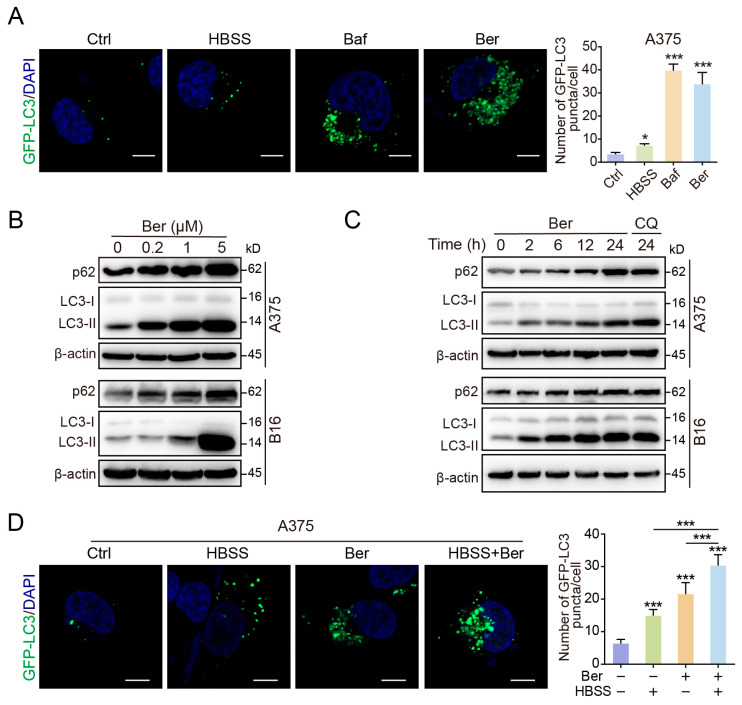
Ber inhibits autophagic flux in melanoma cells. (**A**) Fluorescence microscopy images and quantitative analysis of GFP-LC3 puncta in A375 cells transfected with a GFP-LC3 plasmid, followed by treatment with Ber (5 µM), bafilomycin A1 (Baf, 20 nM) for 24 h, or starvation using Hank’s Balanced Salt Solution (HBSS) for 6 h. Scale bar: 20 µm. (**B**) Western blot analysis showing the levels of LC3-I/II and p62 proteins in A375 and B16 cells treated with Ber at indicated concentrations for 24 h. (**C**) Time-course analysis of LC3-I/II and p62 degradation in A375 cells treated with Ber (5 µM) over different durations (0, 2, 6, 12, 24 h). (**D**) Evaluation of autophagosome formation in A375 cells transfected with GFP-LC3 and treated with or without Ber (5 µM) under starvation conditions (HBSS for 6 h). GFP-LC3 puncta, indicative of autophagosome accumulation, were assessed via fluorescence microscopy. Scale bar: 20 µm. Data are presented as mean ± SD. Statistical significance is indicated by asterisks (* *p* < 0.05, *** *p* < 0.001). Chloroquine, CQ.

In our studies, we noted that Ber elevated MHC-I expression in tumor cells by impeding autophagy. To assess Ber’s impact on autophagy, we first quantified LC3-positive puncta, indicative of autophagosomes, in A375 cells transfected with GFP-LC3. Treatment with Ber (5 µM for 24 h) increased the number of LC3-positive puncta, an effect comparable to that induced by Hank’s Balanced Salt Solution (HBSS, commonly used to induce autophagy via starvation) and the autophagy inhibitor bafilomycin A1 (Baf), suggesting that Ber modulates autophagic processes (Figure 4A). We further investigated the conversion of LC3. During autophagy, cytoplasmic LC3-I converts into membrane-bound LC3-II, which associates with autophagosome membranes and is subsequently degraded in autolysosomes [23]. We observed that LC3-II levels in A375 and B16 cells increased in a time- and dose-dependent manner following Ber treatment (Figure 4B,C), indicating an accumulation of autophagosomes.

To differentiate whether this accumulation was due to increased formation or decreased degradation of autophagosomes, we measured levels of p62. p62 binds ubiquitinated proteins and forms complexes with LC3-II that are degraded in autolysosomes; thus, elevated p62 levels typically suggest disrupted autophagy [24]. We found that Ber treatment caused a concentration-dependent increase in p62 levels (Figure 4B,C), implying that the augmentation in autophagosomes was due to impaired degradation, not increased formation. In alignment with the increases in LC3-II and p62 induced by Ber, we documented a significant rise in LC3-positive puncta in A375 cells transfected with GFP-LC3. Given our previous observations, this likely results from the accumulation of autophagosomes attributable to hindered autophagic degradation. While HBSS treatment alone increased the number of LC3-positive puncta through enhanced autophagosome formation and efficient autophagic flux, the addition of Ber led to a further increase in these puncta (Figure 4D). This finding supports the notion that Ber disrupts the otherwise smooth autophagic flux, leading to an accumulation of undegraded autophagosomes.

### 3.5. Ber Inhibits Late-Stage Autophagy in Melanoma Cells by Suppressing Lysosomal Acidification

**Figure 5:**
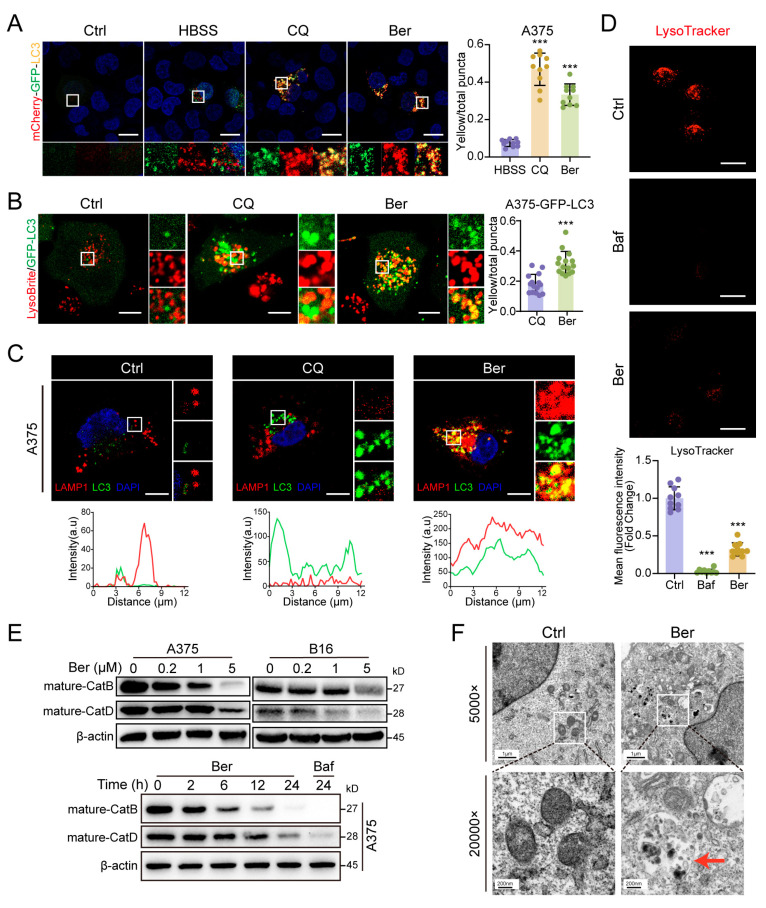
Ber suppresses late-stage autophagy in melanoma cells by inhibiting lysosomal acidification. (**A**) Fluorescence microscopy analysis of A375 cells transiently transfected with mCherry-GFP-LC3, treated with HBSS (starvation) for 6 h, chloroquine (CQ, 20 μM), or Ber (5 μM). Scale bar: 20 μm. Cells treated with HBSS and CQ served as positive controls for starvation and autophagy inhibition, respectively. (**B**) Confocal microscopy images of A375 cells transfected with GFP-LC3 plasmids (green) to label autophagosomes, treated with Ber (10 μM) or CQ (20 μM) for 24 h. Lysosomes were stained with LysoBrite™ red. Yellow fluorescence indicates colocalization of lysosomes and autophagosomes. Scale bar: 20 μm. (**C**) A375 cells transfected with the hLAMP1-mCherry plasmid were treated with Ber (5 μM) or chloroquine (CQ, 20 μM) for 24 h. After immunostaining for LC3-I/II (green), images were captured using confocal microscopy, and the degree of colocalization between hLAMP1-mCherry (red) and LC3 (green) was quantified using ImageJ software. Yellow fluorescence indicates the colocalization of lysosomes and autophagosomes. The red and green traces in the figure represent the arbitrary units (a.u.) of red and green intensities, respectively, within the rectangular region highlighted in the magnified image. Scale bar: 20 μm. (**D**) Representative images of LysoTracker Red staining in A375 cells treated with Ber (5 μM) or bafilomycin A1 (Baf, 50 nM). Quantification of LysoTracker Red fluorescence intensity indicated a decrease in lysosomal acidity following treatment with Ber or Baf. Scale bar: 20 μm. (**E**) Western blot analysis of cathepsin maturation in A375 and B16 cells treated with varying concentrations of Ber and different durations of Ber (5 μM) or Baf (50 nM). (**F**) Electron microscopy of A375 cells treated with Ber (5 μM) for 24 h. Autolysosomes are indicated by red arrowheads. *** *p* < 0.001 indicate levels of statistical significance.

To investigate whether Ber affects the degradation stage of autophagy, we transfected A375 cells with an mCherry-GFP-LC3B tandem reporter, which differentiates autophagosomes from autolysosomes based on their distinct fluorescence. Typically, autophagy induction by starvation (HBSS) increases red fluorescence due to GFP quenching in acidic environments, while disruptions in autophagosome–lysosome fusion or lysosomal function result in persistent yellow fluorescence. Treatment with Ber (5 µM, 24 h) significantly increased the presence of yellow puncta, similar to the effect of the autophagy inhibitor chloroquine (CQ), indicating an impairment in the late stages of autophagic flux (Figure 5A). To determine whether Ber inhibits autophagic flux by affecting autophagosome–lysosome fusion or by impairing autolysosomal degradation, we analyzed the colocalization of GFP-LC3 (autophagosomes) and LysoBrite™ Red (a marker for lysosomes or autolysosomes) in GFP-LC3-transfected A375 cells. CQ treatment led to the accumulation of GFP-LC3 puncta with minimal colocalization with lysosomes, indicating disrupted fusion. In contrast, Ber treatment resulted in extensive colocalization, producing numerous yellow puncta (Figure 5B), suggesting that autophagosomes successfully fused with lysosomes.

To further confirm this, we transfected A375 cells with the hLAMP1-mCherry plasmid to directly observe the colocalization of LAMP1 (a lysosomal marker) and LC3 (an autophagosomal marker). Unlike CQ treatment, Ber treatment led to significant colocalization of LAMP1 and LC3 (Figure 5C), further supporting intact autophagosome–lysosome fusion. Additionally, LysoTracker staining, a well-established method for detecting lysosomal acidification, demonstrated that Ber significantly reduced lysosomal acidity (Figure 5D). Ber also decreased the levels of mature lysosomal enzymes such as cathepsin B and cathepsin D, both of which depend on normal lysosomal acidification (Figure 5E). Moreover, electron microscopy of Ber-treated A375 cells revealed the presence of autolysosomes formed after autophagosome–lysosome fusion (Figure 5F). These findings provide strong evidence that Ber does not inhibit autophagosome–lysosome fusion but rather suppresses lysosomal acidification, thereby inhibiting late-stage autophagy in melanoma cells.

### 3.6. Ber Suppresses Melanoma Tumor Growth in Mice by Enhancing CD8^+^ T Cell Infiltration 

**Figure 6:**
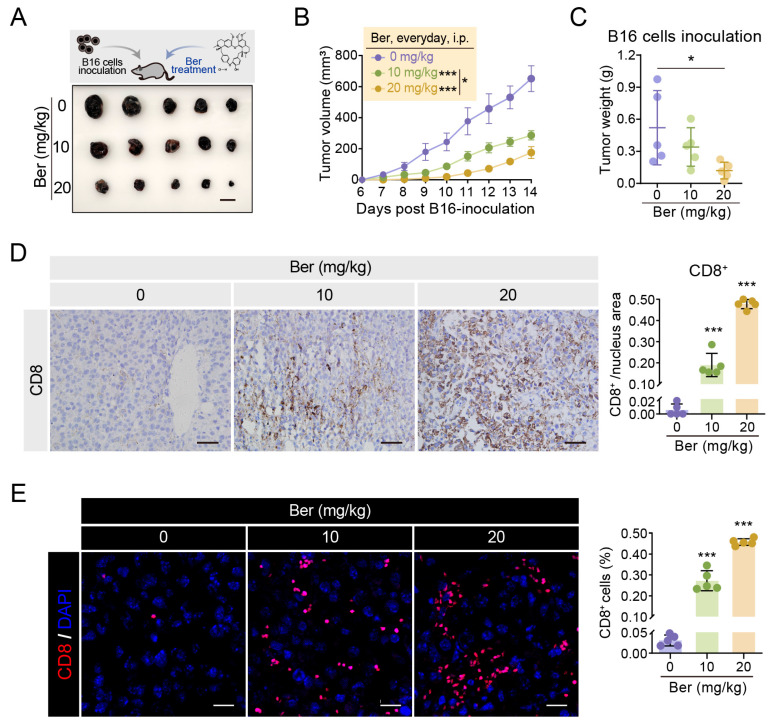
Ber suppresses melanoma tumor growth and enhances CD8^+^ T cell infiltration in mice. (**A**) Subcutaneous tumors were established in C57BL/6 mice by injecting B16 melanoma cells into the right axillary region. Mice were randomly assigned to three groups and received intraperitoneal injections of Ber at specified doses for 14 days. Post-treatment, the tumors were surgically excised for analysis (*n* = 5 per group). Scale bar: 1 cm. (**B**) Graph showing the progression of tumor growth in each group over the treatment period, with values expressed as mean ± SEM (*n* = 5 per group). (**C**) Tumor weights were measured to assess the efficacy of Ber in inhibiting tumor growth across the treatment groups. (**D**) Representative immunohistochemistry images displaying CD8^+^ T cell staining in tumor tissues from each experimental group, illustrating the degree of immune cell infiltration. Scale bar: 50 μm. (**E**) Confocal immunofluorescent images and corresponding quantification of CD8^+^ T cells in B16 melanoma tissues from mice. Scale bar, 40 μm. * *p* < 0.05, *** *p* < 0.001 indicates levels of statistical significance.

To evaluate the effects of Ber on melanoma tumor growth, B16 melanoma cells were subcutaneously injected into the right axillary region of C57BL/6 mice to establish subcutaneous tumors. The mice were randomly assigned into three groups: Ber-treated at 0 mg/kg, Ber-treated at 10 mg/kg, and Ber-treated at 20 mg/kg. Tumor volume was measured daily (*n* = 5 per group) (Figure 6B). After 14 days, the animals were euthanized, and tumors were harvested and weighed (Figure 6A,C). Notably, a significant suppression of tumor growth was observed in both Ber-treated groups. Immunohistochemical and immunofluorescent analyses of CD8 staining in subcutaneous B16 tumors from mice were conducted to assess the infiltration of CD8^+^ T cells within the tumor tissues. The results demonstrated that Ber treatment significantly increased CD8^+^ T cell infiltration in melanoma tumors (Figure 6D,E). These findings demonstrate that Ber not only inhibits melanoma progression but also enhances the immune response within the tumor microenvironment, potentially through the recruitment and activation of cytotoxic T cells.

## 4. Discussion

We report that Ber upregulates MHC-I expression on the surface of melanoma cells, thereby enhancing antigen presentation and the subsequent recognition and elimination of tumor cells by CD8^+^ T cells, circumventing tumor immune escape. Given the known role of autophagy in tumor immune evasion [25], we hypothesized that Ber might augment MHC-I protein expression by inhibiting autophagy, thus boosting antigen presentation. Our findings confirm this hypothesis, showing that autophagy inhibition raises MHC-I protein levels on melanoma cells, and further increases in MHC-I expression are not observed with Ber treatment following Atg4b mutation. This lack of an additive effect suggests that Ber’s primary mechanism involves autophagy inhibition.

Further investigation revealed that Ber inhibits autophagy by suppressing lysosomal acidification within tumor cells. MHC (major histocompatibility complex) molecules play a crucial role in enabling T cells to specifically recognize and target cells. We focus particularly on MHC-I, as CD8^+^ T cells recognize antigens presented by MHC-I on the surface of tumor cells, thereby initiating immune responses [26,27]. A reduction in surface MHC-I expression can allow tumor cells to evade recognition by CD8^+^ T cells, facilitating immune evasion. Previous studies corroborate our findings; for instance, Angel M. Garcia-Lora et al. demonstrated that MHC-I downregulation enhances melanoma cell carcinogenicity, proliferation, migration, and invasion in vivo and in vitro [26]. Similarly, Xiaowei Liu et al. reported a significant association between MHC-I antigen presentation loss in melanoma cells and resistance to PD-1 blockade [27]. Thus, enhancing MHC-I expression on tumor cells is crucial for improving the efficacy of melanoma therapies.

In our study, we treated two melanoma cell lines with varying concentrations of Ber and observed a significant increase in surface MHC-I expression (Figure 1B). We also noted a rise in total cellular MHC-I protein levels (Figure 1D). Notably, Ber did not affect MHC-I transcription in either melanoma cell line (Figure S1), leading us to speculate that Ber modulates MHC-I degradation pathways. Proteins may be degraded through the proteasome pathway or the autophagy–lysosome pathway. Interestingly, when autophagy was inhibited via a (Dox)-inducible Atg4b mutation and the cells were subsequently treated with Ber, there was no further increase in MHC-I (H-2K^b^) levels (Figure 3C,D), and H-2K^b^-SIINFEKL presentation was not additionally enhanced (Figure 3E,F). These findings suggest that Ber primarily enhances MHC-I expression and antigen presentation in melanoma cells through the inhibition of autophagy.

The 257-264 peptide of the ovalbumin (OVA) protein is known to be presented by MHC-I. In OVA-expressing B16 cells, it forms complexes with MHC-I on the cell surface. These complexes can be specifically recognized and bound by the antibody, marking cells that have undergone OVA antigen presentation. Flow cytometry analysis showed that Ber treatment increased antigen presentation levels in a concentration-dependent manner (Figure 1E). Increasing antigen presentation levels theoretically enhances the specific killing by CD8^+^ T cells. For example, Arul M. Chinnaiyan et al. demonstrated that augmenting tumor-specific MHC-I expression increases the number of functional CD8^+^ T cells within the tumor and slows tumor progression in several syngeneic *mouse* models [28]. The T cell receptor (TCR) on CD8^+^ T cells recognizes and binds to the tumor antigen-MHC-I complex on the cell surface, triggering the release of cytotoxic substances such as perforin, tumor necrosis factor, and interferon to kill tumor cells [29]. We further validated these findings by co-culturing Ber-treated B16-OVA cells with CD8^+^ T cells from OT-I mice, which recognize the OVA protein’s 257-264 peptide presented by MHC-I. Post-treatment, we observed an enhanced killing capacity of T cells (Figure 2B,D), and ELISA assays confirmed a significant increase in IFN-γ release in the co-culture system, indicating CD8^+^ T cell activation. In vivo experiments also confirmed that Ber significantly increased CD8^+^ T cell infiltration in melanoma tumors and inhibited tumor growth (Figure 6), substantiating Ber’s potential as an immunotherapeutic agent in melanoma treatment.

Our hypothesis posits that Ber enhances MHC-I expression in tumor cells by inhibiting autophagy, a process increasingly recognized as a pivotal factor in tumor immune evasion [25,30]. For instance, Ravi K. Amaravadi et al. observed that stage IV melanoma patients exhibiting poor responses to dacarbazine and sorafenib chemotherapy had elevated autophagy levels in tumor cells [31]. Moreover, research indicates that melanoma resistance to chemotherapy may be associated with autophagy induction triggered by Wnt5A expression [32]. Autophagy typically progresses from the formation of a pre-autophagosomal structure (PAS) upon sensing cellular stress, to the recruitment and assembly of autophagy-related proteins (ATGs) that form autophagosomes. These autophagosomes eventually fuse with lysosomes to create autolysosomes, where their contents are degraded by enzymes [33]. Initially, autophagy may suppress tumor formation by mitigating oncogenic stressors such as chronic tissue damage and genomic instability [34,35]. However, in advanced tumors, autophagy may help cancer cells survive under adverse conditions like hypoxia and nutrient deprivation [36,37]. Given that most cancers are diagnosed at advanced stages, targeting autophagy could provide therapeutic benefits.

Ber has been reported as an autophagy inhibitor, with Fu et al. showing that Ber suppresses the accumulation of autophagosomes in breast cancer cells by upregulating BNIP3 and blocking autophagosome–lysosome fusion [38]. In contrast, our study reveals that in melanoma cells, Ber operates through a distinct mechanism—specifically, by inhibiting lysosomal acidification, which in turn suppresses late-stage autophagy. Our LysoTracker staining, a well-established method for detecting lysosomal acidification, demonstrated that Ber treatment significantly reduced lysosomal acidity (Figure 5D). Additionally, Ber decreased the levels of mature lysosomal enzymes such as cathepsin B and cathepsin D, both of which are dependent on normal lysosomal acidification (Figure 5E). These findings strongly suggest that Ber inhibits autophagy by suppressing lysosomal acidification, thereby impairing the final stage of autophagic flux. To further elucidate whether Ber inhibits autophagic flux by affecting autophagosome–lysosome fusion, we conducted a co-localization analysis using GFP-LC3 and LysoBrite™ Red, a dye less sensitive to acidity changes than LysoTracker. Our analysis revealed that GFP-LC3-positive puncta in Ber-treated cells largely co-localized with lysosomes (Figure 5B). Additionally, we transfected A375 melanoma cells with the hLAMP1-mCherry plasmid to mark lysosomes with red fluorescence, while autophagosomes were marked with green fluorescence via immunofluorescence staining of LC3. Unlike chloroquine (CQ) treatment, Ber treatment resulted in significant co-localization of LAMP1 (lysosomes) and LC3 (autophagosomes) (Figure 5C). Furthermore, electron microscopy of Ber-treated A375 cells revealed the presence of autolysosomes, formed after the fusion of autophagosomes and lysosomes (Figure 5F). These results provide clear evidence that Ber does not prevent autophagosome–lysosome fusion but rather inhibits lysosomal acidification, thereby suppressing late-stage autophagy in melanoma cells. While our findings differ from those of Fu et al. [38], this discrepancy may be attributed to the distinct cellular contexts of different cancer types. Nevertheless, our study significantly advances the understanding of Ber’s mechanism in melanoma cells and supports the broader conclusion that Ber inhibits late-stage autophagy.

Recent interest in the therapeutic potential of natural products for drug development has grown significantly. Ber, derived from the Chinese medicinal herbs Coptis chinensis (Chinese goldthread) and Berberis spp. (barberry), is primarily known for its anti-inflammatory properties [15,39]. Its hydrochloride form, particularly, has been explored for anticancer activities. Notable studies include Han C et al., who demonstrated that Ber modulates the ROS/NF-κB signaling pathway to exert anti-tumor effects in bladder cancer [40]; Bingren Hu et al., who highlighted its efficacy against pancreatic cancer [41]; Parhi et al., who observed inhibitory effects on the growth of B16 melanoma in a C57BL/6 *mouse* model [42]; and Fu et al., who reported that Ber suppresses the accumulation of autophagosomes in breast cancer cells [38]. Our study adds to this field by demonstrating that Ber, as a late-stage autophagy inhibitor, suppresses autophagy in melanoma cells by inhibiting lysosomal acidification. This inhibition significantly enhances MHC-I-mediated antigen presentation, thereby boosting the specific immune killing of tumors by CD8^+^ T cells. 

Currently, only chloroquine and its derivative hydroxychloroquine are FDA-approved for this purpose, primarily due to safety concerns with other compounds [43]. Given the historical use of Ber and its established safety profile [44], it emerges as a promising candidate for the development of safer and more effective autophagy inhibitors. In a therapeutic landscape where options for clinical autophagy inhibitors are scarce, Ber could potentially augment tumor cell eradication when used alongside conventional anticancer drugs through its modulation of autophagy. In summary, Ber’s ability to inhibit lysosomal acidification disrupts autophagic flux in melanoma cells. This disruption enhances MHC-I-mediated antigen presentation, thereby improving the efficacy of CD8^+^ T cell-mediated tumor cell destruction. This mechanism highlights Ber’s potential as a valuable adjunct in melanoma therapy and underscores the broader applicability of natural products in medical research.

## 5. Conclusions

Our research demonstrates that Ber effectively inhibits late-stage autophagy by targeting lysosomal acidification, enhancing MHC-I-mediated antigen presentation in melanoma cells. This improvement in antigen presentation facilitates better recognition and elimination of tumor cells by CD8^+^ T cells. These findings suggest that Ber has potential as an adjuvant therapy in melanoma treatment.

## Data Availability

The original contributions presented in the study are included in the article/Supplementary Material. Further inquiries can be directed to the corresponding author.

## Supplementary Materials

The following supporting information can be downloaded at: https://www.mdpi.com/article/10.3390/cells13181537/s1, Figure S1: Relative mRNA expression levels of MHC-I genes. (A) mRNA expression of *HLA-A* and *HLA-B* genes in A375 cells measured by RT-qPCR after 24-h treatment with varying concentrations of Ber. (B) mRNA expression of *H-2K1* and *H-2D1* genes in B16 cells under the same treatment conditions. No statistically significant differences were observed between the groups (ns). Berbamine hydrochloride: Ber. Figure S2: Cell viability assay of B16 cells. Cells were treated with various concentrations of Ber for 24 h, and viability was assessed using the CCK8 assay. No statistically significant difference was observed in cell viability between the control group and the Ber-treated groups (ns). Berbamine hydrochloride: Ber. Table S1: The sequences of qRT-PCR primers in this study.

## References

[1] Sung H., Ferlay J., Siegel R.L., Laversanne M., Soerjomataram I., Jemal A., Bray F. (2021). Global cancer statistics 2020: Globocan estimates of incidence and mortality worldwide for 36 cancers in 185 countries. CA Cancer J. Clin..

[2] Balch C.M., Gershenwald J.E., Soong S.J., Thompson J.F., Atkins M.B., Byrd D.R., Buzaid A.C., Cochran A.J., Coit D.G., Ding S. (2009). Final version of 2009 ajcc melanoma staging and classification. J. Clin. Oncol. Off. J. Am. Soc. Clin. Oncol..

[3] Siegel R.L., Miller K.D., Wagle N.S., Jemal A. (2023). Cancer statistics, 2023. CAA Cancer J. Clin..

[4] Cao J., Yan Q. (2020). Cancer epigenetics, tumor immunity, and immunotherapy. Trends Cancer.

[5] Li J., Wang K., Yang C., Zhu K., Jiang C., Wang M., Zhou Z., Tang N., Wang Q., Wang S. (2023). Tumor-associated macrophage-derived exosomal linc01232 induces the immune escape in glioma by decreasing surface mhc-i expression. Adv. Sci..

[6] Duan Y., Tian X., Liu Q., Jin J., Shi J., Hou Y. (2021). Role of autophagy on cancer immune escape. Cell Commun. Signal. CCS.

[7] Gao L., Chen Y. (2021). Autophagy controls programmed death-ligand 1 expression on cancer cells (review). Biomed. Rep..

[8] Merkley S.D., Chock C.J., Yang X.O., Harris J., Castillo E.F. (2018). Modulating t cell responses via autophagy: The intrinsic influence controlling the function of both antigen-presenting cells and t cells. Front. Immunol..

[9] Wang K., Liu X., Liu Q., Ho I.H., Wei X., Yin T., Zhan Y., Zhang W., Zhang W., Chen B. (2020). Hederagenin potentiated cisplatin- and paclitaxel-mediated cytotoxicity by impairing autophagy in lung cancer cells. Cell Death Dis..

[10] Chude C.I., Amaravadi R.K. (2017). Targeting autophagy in cancer: Update on clinical trials and novel inhibitors. Int. J. Mol. Sci..

[11] Wang C., Hu Q., Shen H.M. (2016). Pharmacological inhibitors of autophagy as novel cancer therapeutic agents. Pharmacol. Res..

[12] Mauvezin C., Nagy P., Juhász G., Neufeld T.P. (2015). Autophagosome-lysosome fusion is independent of v-atpase-mediated acidification. Nat. Commun..

[13] Mauthe M., Orhon I., Rocchi C., Zhou X., Luhr M., Hijlkema K.J., Coppes R.P., Engedal N., Mari M., Reggiori F. (2018). Chloroquine inhibits autophagic flux by decreasing autophagosome-lysosome fusion. Autophagy.

[14] Shi T.T., Yu X.X., Yan L.J., Xiao H.T. (2017). Research progress of hydroxychloroquine and autophagy inhibitors on cancer. Cancer Chemother. Pharmacol..

[15] Zhu J., Huang L., Gao F., Jian W., Chen H., Liao M., Qi W. (2022). Berbamine hydrochloride inhibits african swine fever virus infection in vitro. Molecules.

[16] Yi D., Li Q., Wang H., Lv K., Ma L., Wang Y., Wang J., Zhang Y., Liu M., Li X. (2022). Repurposing of berbamine hydrochloride to inhibit ebola virus by targeting viral glycoprotein. Acta Pharm. Sin. B.

[17] Zhang X.P., Zhang X., Yang H.J., Zou D.H., He X.M., Yu X.F., Li Y.F. (2015). Treatment of chemotherapy related leukocytopenia by oral administration of multiple leucogenic drugs combined with g-csf: An experimental study. Zhongguo Zhong Xi Yi Jie He Za Zhi Zhongguo Zhongxiyi Jiehe Zazhi = Chin. J. Integr. Tradit. West. Med..

[18] Zhan Y., Chen Q., Song Y., Wei X., Zhao T., Chen B., Li C., Zhang W., Jiang Y., Tan Y. (2023). Berbamine hydrochloride inhibits lysosomal acidification by activating nox2 to potentiate chemotherapy-induced apoptosis via the ros-mapk pathway in human lung carcinoma cells. Cell Biol. Toxicol..

[19] Campesato L.F., Budhu S., Tchaicha J., Weng C.-H., Gigoux M., Cohen I.J., Redmond D., Mangarin L., Pourpe S., Liu C. (2020). Blockade of the ahr restricts a treg-macrophage suppressive axis induced by l-kynurenine. Nat. Commun..

[20] Fonderflick L., Baudu T., Adotévi O., Guittaut M., Adami P., Delage-Mourroux R.J.C. (2022). The atg8 family proteins gabarap and gabarapl1 target antigen to dendritic cells to prime cd4+ and cd8+ t cells. Cells.

[21] N’Diaye E.N., Kajihara K.K., Hsieh I., Morisaki H., Debnath J., Brown E.J. (2009). Plic proteins or ubiquilins regulate autophagy-dependent cell survival during nutrient starvation. EMBO Rep..

[22] Yamamoto K., Venida A., Yano J., Biancur D.E., Kakiuchi M., Gupta S., Sohn A.S.W., Mukhopadhyay S., Lin E.Y., Parker S.J. (2020). Autophagy promotes immune evasion of pancreatic cancer by degrading mhc-i. Nature.

[23] Tanida I., Ueno T., Kominami E. (2004). Lc3 conjugation system in mammalian autophagy. Int. J. Biochem. Cell Biol..

[24] Lamark T., Svenning S., Johansen T. (2017). Regulation of selective autophagy: The p62/sqstm1 paradigm. Essays Biochem..

[25] Yang W., Chen H., Li G., Zhang T., Sui Y., Liu L., Hu J., Wang G., Chen H., Wang Y. (2023). Caprin-1 influences autophagy-induced tumor growth and immune modulation in pancreatic cancer. J. Transl. Med..

[26] Garrido C., Paco L., Romero I., Berruguilla E., Stefansky J., Collado A., Algarra I., Garrido F., Garcia-Lora A.M. (2012). Mhc class i molecules act as tumor suppressor genes regulating the cell cycle gene expression, invasion and intrinsic tumorigenicity of melanoma cells. Carcinogenesis.

[27] Yu J., Wu X., Song J., Zhao Y., Li H., Luo M., Liu X. (2022). Loss of mhc-i antigen presentation correlated with immune checkpoint blockade tolerance in mapk inhibitor-resistant melanoma. Front. Pharmacol..

[28] Bao Y., Qiao Y., Choi J.E., Zhang Y., Mannan R., Cheng C., He T., Zheng Y., Yu J., Gondal M. (2023). Targeting the lipid kinase pikfyve upregulates surface expression of mhc class i to augment cancer immunotherapy. Proc. Natl. Acad. Sci. USA.

[29] Lees J.R. (2020). Cd8+ t cells: The past and future of immune regulation. Cell. Immunol..

[30] Xing S.L., Li G.X., Ding J.X., Wu G.H., Qin L. (1987). Pharmacological study of infection of sansheng. Zhong Yao Tong Bao (Beijing, China 1981).

[31] Ma X.H., Piao S., Wang D., McAfee Q.W., Nathanson K.L., Lum J.J., Li L.Z., Amaravadi R.K. (2011). Measurements of tumor cell autophagy predict invasiveness, resistance to chemotherapy, and survival in melanoma. Clin. Cancer Res. Off. J. Am. Assoc. Cancer Res..

[32] Ndoye A., Budina-Kolomets A., Kugel C.H., Webster M.R., Kaur A., Behera R., Rebecca V.W., Li L., Brafford P.A., Liu Q. (2017). Atg5 mediates a positive feedback loop between wnt signaling and autophagy in melanoma. Cancer Res..

[33] Parzych K.R., Klionsky D.J. (2014). An overview of autophagy: Morphology, mechanism, and regulation. Antioxid. Redox Signal..

[34] Sakurai T., He G., Matsuzawa A., Yu G.Y., Maeda S., Hardiman G., Karin M. (2008). Hepatocyte necrosis induced by oxidative stress and il-1 alpha release mediate carcinogen-induced compensatory proliferation and liver tumorigenesis. Cancer Cell.

[35] Sun B., Karin M. (2008). Nf-kappab signaling, liver disease and hepatoprotective agents. Oncogene.

[36] Suzuki S.W., Onodera J., Ohsumi Y. (2011). Starvation induced cell death in autophagy-defective yeast mutants is caused by mitochondria dysfunction. PLoS ONE.

[37] Onodera J., Ohsumi Y. (2005). Autophagy is required for maintenance of amino acid levels and protein synthesis under nitrogen starvation. J. Biol. Chem..

[38] Fu R., Deng Q., Zhang H., Hu X., Li Y., Liu Y., Hu J., Luo Q., Zhang Y., Jiang X. (2018). A novel autophagy inhibitor berbamine blocks snare-mediated autophagosome-lysosome fusion through upregulation of BNIP3. Cell Death Dis..

[39] Ishigami J. (1986). Problems of complicated urinary tract infections and infections with multiple pathogens. Nihon Rinsho. Jpn. J. Clin. Med..

[40] Han C., Wang Z., Chen S., Li L., Xu Y., Kang W., Wei C., Ma H., Wang M., Jin X. (2021). Berbamine suppresses the progression of bladder cancer by modulating the ros/nf-κb axis. Oxidative Med. Cell. Longev..

[41] Hu B., Yang Y., Tu J., Cai H., Yang S., Chen X., Chen G. (2023). Berbamine exerts an anti-oncogenic effect on pancreatic cancer by regulating wnt and DNA damage-related pathways. Anti-Cancer Agents Med. Chem..

[42] Parhi P., Suklabaidya S., Kumar Sahoo S.J.S.R. (2017). Enhanced anti-metastatic and anti-tumorigenic efficacy of berbamine loaded lipid nanoparticles in vivo. Sci. Rep..

[43] Mahalingam D., Mita M., Sarantopoulos J., Wood L., Amaravadi R.K., Davis L.E., Mita A.C., Curiel T.J., Espitia C.M., Nawrocki S.T. (2014). Combined autophagy and hdac inhibition: A phase i safety, tolerability, pharmacokinetic, and pharmacodynamic analysis of hydroxychloroquine in combination with the hdac inhibitor vorinostat in patients with advanced solid tumors. Autophagy.

[44] Mokhber-Dezfuli N., Saeidnia S., Gohari A.R., Kurepaz-Mahmoodabadi M. (2014). Phytochemistry and pharmacology of berberis species. Pharmacogn. Rev..

