# Supplementary material for: Inhibition of Autophagy by Berbamine Hydrochloride Mitigates Tumor Immune Escape by Elevating MHC-I in Melanoma Cells
Source: Cells. 2024 Sep 13;13(18):1537. doi: 10.3390/cells13181537 (PMC11430705; doi:10.3390/cells13181537)
Supplement: Supplementary file 1 [file cells-13-01537-s001.zip › cells-3070520-supplementary.pdf]

Inhibition of Autophagy by Berbamine Hydrochloride Mitigates Tumor Immune Escape by Elevating MHC-I in Melanoma Cells

Jinhuan Xian [1,2,†], Leilei Gao [1,2,†], Zhenyang Ren [1,3], Yanjun Jiang [4], Junjun Pan [1], Zheng Ying [1], Zhenyuan Guo [1], Qingsong Du [1], Xu Zhao [1], He Jin [1,2], Hua Yi [1,2], Jieying Guan [1,2,*] and Shan Hu [1,2,*]

[1] Research Center of Integrative Medicine, School of Basic Medical Sciences, Guangzhou University of Chinese Medicine, Guangzhou 510006, China; jinhuanxian@stu.gzucm.edu.cn (J.X.); gaoleilei@stu.gzucm.edu.cn (L.G.); renzhenyang@stu.gzucm.edu.cn (Z.R.); panjunjun1@oncolab.cn (J.P.); yingzheng@oncolab.cn (Z.Y.); guozhenyuan@oncolab.cn (Z.G.); duqingsong@oncolab.cn (Q.D.); zhaoxu@oncolab.cn (X.Z.); jinhe188@gzucm.edu.cn (H.J.); yingying020@gzucm.edu.cn (H.Y.)

[2] Department of Pathology and Pathophysiology, Guangzhou University of Chinese Medicine, Guangzhou 510006, China

[3] Department of Biochemistry, Guangzhou University of Chinese Medicine, Guangzhou 510006, China

[4] Department of Anaesthesia and Intensive Care, The Chinese University of Hong Kong, Hong Kong 999077, China; 1155149754@link.cuhk.edu.hk

* Correspondence: cecilia_100@gzucm.edu.cn (J.G.); hushan@gzucm.edu.cn (S.H.)

† These authors contributed equally to this work.

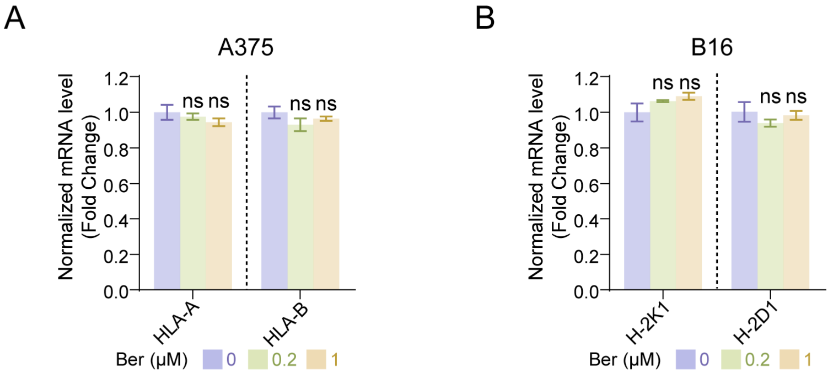

Figure S1. Relative mRNA expression levels of MHC-I genes. (A) mRNA expression of HLA-A and HLA-B genes in A375 cells measured by RT-qPCR after 24-hour treatment with varying concentrations of Ber. (B) mRNA expression of H-2K1 and H-2D1 genes in B16 cells under the same treatment conditions. No statistically significant differences were observed between the groups (ns). Berbamine hydrochloride: Ber.

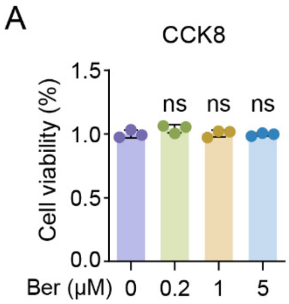

Figure S2. Cell viability assay of B16 cells. Cells were treated with various concentrations of Ber for 24 hours, and viability was assessed using the CCK8 assay. No statistically significant difference was observed in cell viability between the control group and the Ber-treated groups (ns). Berbamine hydrochloride: Ber.

Table S1. The sequences of qRT-PCR primers in this study.

Primer	Sequences
**HLA-A**	Forward: 5′-AAAAGGAGGGAGTTACACTCAGG-3'
	Reverse: 5′-GCTGTGAGGGACACATCAGAG-3'
**HLA-B**	Forward: 5′-GGGATGGCGAGGACCAAAC-3'
	Reverse: 5′-ACAGCTCCGATGACCACAAC-3'
**ACTB**	Forward: 5'-GCCGTCTTCCCCTCCATC-3'
	Reverse: 5'-CTGACCCATGCCCACCAT-3'
**H-2K1**	Forward: 5'-GCTGGTGAAGCAGAGAGACTCAG-3'
	Reverse: 5'- GGTGACTTTATCTTCAGGTCTGCT-3'
**H-2D1**	Forward: 5'- AGTGGTGCTGCAGAGCATTACAA-3'
	Reverse: 5'-GGTGACTTCACCTTTAGATCTGGG-3'
**Actb**	Forward: 5'-GGCTGTATTCCCCTCCATCG-3'
	Reverse: 5'-CCAGTTGGTAACAATGCCATGT-3'
